# Molecular Modeling Applied to Nucleic Acid-Based Molecule Development

**DOI:** 10.3390/biom8030083

**Published:** 2018-08-27

**Authors:** Arne Krüger, Flávia M. Zimbres, Thales Kronenberger, Carsten Wrenger

**Affiliations:** 1 Unit for Drug Discovery, Department of Parasitology, Institute of Biomedical Sciences, University of São Paulo, São Paulo, SP 05508-000, Brazil; krueger.arne@icb.usp.br; 2 Department of Biochemistry and Molecular Biology and Center for Tropical and Emerging Global Diseases, University of Georgia, Athens, GA 30602, USA; flavia.zimbres@uga.edu; 3 Department of Internal Medicine VIII, University Hospital of Tübingen, 72076 Tübingen, Germany; kronenberger7@gmail.com

**Keywords:** aptamers, docking, molecular dynamics, nucleic acids

## Abstract

Molecular modeling by means of docking and molecular dynamics (MD) has become an integral part of early drug discovery projects, enabling the screening and enrichment of large libraries of small molecules. In the past decades, special emphasis was drawn to nucleic acid (NA)-based molecules in the fields of therapy, diagnosis, and drug delivery. Research has increased dramatically with the advent of the SELEX (systematic evolution of ligands by exponential enrichment) technique, which results in single-stranded DNA or RNA sequences that bind with high affinity and specificity to their targets. Herein, we discuss the role and contribution of docking and MD to the development and optimization of new nucleic acid-based molecules. This review focuses on the different approaches currently available for molecular modeling applied to NA interaction with proteins. We discuss topics ranging from structure prediction to docking and MD, highlighting their main advantages and limitations and the influence of flexibility on their calculations.

## 1. Introduction—Nucleic Acids as a Class of Drugs and Biomarkers

In drug discovery and delivery, DNA and RNA play an important role as therapeutics, biomarkers, and ligands for targeted delivery [1]. One technique that was especially developed over the past decades and gained increasing importance is SELEX (systematic evolution of ligands by exponential enrichment). The SELEX technique was developed and described for the first time by two independent laboratories in 1990 [2,3]. Starting with a library of randomly generated oligonucleotides composed of a central variable region of random nucleotides flanked by constant sequence regions (priming regions), the entire sequences can easily be amplified via conventional PCR. The product can then be readily selected against a specific target using positive and negative selection cycles. DNA or RNA oligonucleotides, which are a result of several selection cycles, are called aptamers and can bind with high specificity and affinity to their targets. Aptamers represent interesting alternatives to antibodies and are used in a variety of applications, as already mentioned above [1]. Although antibodies and aptamers bear resemblance to each other as dissociation constants at the nano- to picomolar level, aptamers show superior advantages that should be considered. Aptamers (i) can distinguish between isomeric and conformational forms of the same protein [4,5]; (ii) are amplified in vitro without the use of animals and therefore show little batch-to-batch variation; (iii) can be produced within a short time period by low-cost high throughput systems [1]; (iv) offer a broad range of targets, including nonimmunogenic agents like toxins, and do not cause a immunogenic response themselves [6]; (v) are smaller, which allows faster uptake and targeting of otherwise inaccessible structures; (vi) are very stable and resistant to fluctuations in temperature and pH [1]; and (vii) can be conveniently and easily modified in various ways without affecting the binding affinity [7].

Originally used in in vitro experiments, it has been shown that SELEX can also be applied onto more complex targets, e.g., whole cells such as erythrocytes and cancer cells (Cell-SELEX). Therefore, potential targets can be targeted at their native conformation [1,8]. Aptamers are used in a wide variety of applications, including Aptasensors, ribozymes, and DNAzymes, and the interest in new oligonucleotide products has fuelled research in the field of production and process optimization [1]. Thus, the research in the field of functional nucleic acids (NAs) has become of high interest over the last decades [9]. Former examples of nucleic acid (NA)-binding proteins include glutamine tRNA synthetase (GlnRS) and T4 bacteriophage DNA polymerase (gp43), which are also targets of aptamer interaction [9]. However, in recent years, it has been shown that aptamers can also bind to proteins that are normally not involved in NA interaction. In DNA–protein interaction, binding predominantly occurs in the major groove of DNA, although minor groove interactions have also been reported [10,11,12]. Furthermore, interactions of aptamer and protein mainly occur due to hydrogen bonding or electropositive charge–charge interactions and, to a less extent, rely on van der Waals forces and hydrophobic groove contributions [9]. Therefore, a wide range of possible modifications, such as alterations of the base, phosphate group, or sugar ring with functional groups (such as fluoro-(F), amino-(NH_2_) or *O*-methyl-(OCH_3_) groups), could not only increase nuclease resistance [13] but also allow hydrophobic epitopes to be targeted on the surfaces of the proteins, therefore increasing the variety of aptamer targets [14]. For instance, modified NA-ligands have been constructed harboring special 5’-position-modified pyrimidine bases in DNA by attaching functional groups [15,16]. Other approaches have utilized backbone modifications of NA, such as a 1,5-anhydrohexitol nucleic acid, cyclohexenyl nucleic acid, 20-O, 40-C, methylene-b-d-ribonucleic acid, arabino nucleic acid, and 20-fluoro-arabino-nucleic acid [17] (also reviewed by Meek et al. [18]). Available modified nucleotides, for instance, are altered at the 2’-position of ribose, which has been shown to influence RNA structure flexibility [19]. However, functionalized NA molecules are not limited to improve binding properties but could also influence serum stability and pharmacokinetic properties [20]. It is expected that the incorporation of click chemistry-based approaches for the development of new modified nucleotides and the use of newly developed polymerases that accept them will improve the accessibility of functionalized NAs in the market [21,22].

## 2. In Silico Approaches for Structure Prediction and Docking

### 2.1. Systematic Evolution of Ligands by Exponential Enrichment—Alternative Algorithms

Despite the successful cases, SELEX can still be time- and resource-consuming and the success varies, depending—among other factors—on the quality and composition of the applied oligonucleotide library [23]. Therefore, several different approaches have been developed to simplify the whole selection process. Most notable of these are in silico techniques that allow for aptamer design and selection completely from scratch [24,25]. These include the computational design of an aptamer library [26,27] as well as a selection of aptamers against a given target [27,28]. The general problems with these approaches are the lack of random libraries and the necessary calculation power, which is disproportionately high [23,27,28]. Another interesting approach was published in 2016 by Ahirwar and colleagues who tried to design an aptamer to target estrogen receptor alpha (ERα) in silico. Instead of generating a pool of random sequences, they deployed a “bottom-up” method utilizing a natural binding ligand of ERα—estrogen response element—to create aptamers and optimized the binding affinities [29].

An interesting alternative approach was recently presented by a group from Heidelberg trying to circumvent the main flaws of aptamer design and development using experimental and in silico approaches. In 2015, the group participated in the International Genetically Engineered Machine (iGEM) competition and developed software named M.A.W.S., short for Making Aptamers Without SELEX. The algorithm loads the target molecule and creates a bounding box. For each nucleotide, the best conformation is calculated and the position with the lowest entropy is selected as the starting point for the next calculation cycle. Thereby, the algorithm constructs aptamers from scratch without using a given aptamer library against any given target structure [23].

### 2.2. Aptamer Secondary and Tertiary Structure Prediction

The binding affinity and specificity of aptamers derive from their specific secondary and tertiary structures, which allow for the recognition of different target structures [30]. The viability of aptamer application is based on the high flexibility of the (deoxy)ribose-phosphodiester backbone, which possesses a total of six torsion angles/flexible bonds and therefore allows for a wide variety of secondary and tertiary structures [9]. On the other hand, structures are narrowed due to the limitation of only four bases. Therefore, the already mentioned modifications were introduced to broaden the range of structures and NA–protein interactions [16,31].

Nucleic acid modeling must consider the flexibility of the phosphodiester backbone and all possible base pairings, including noncanonical base pairing as well as the influence of hydrophobic interactions and best free energy conformations [32]. These factors pose a great challenge in terms of algorithm approach and, later, computing power. Several algorithms exist to calculate the secondary structures and recognize motifs of short to medium ranged DNA and RNA molecules that apply different approaches, such as MEME Suite [33], MEMERIS [34], GLAM2 [35], mFold [36], and Aptamotif [37], etc. Secondary structures occur as a result of intramolecular nucleotide pairing and are the reason for target–ligand interactions [36]. Among pseudoknots and G-quadruplex, the most common structures are stem-loops, which comprise four different substructures: (i) hairpin loop, (ii) bulge loop, (iii) interior loop, and (iv) multibranch loop that again form more complex structures such as kissing hairpins [1,37]. While some algorithms are physic-based and employ thermodynamics (such as the Turner’s thermodynamics table) or the nearest-neighbor model, e.g., mFold, and only consider canonical base-pairings [36,38,39], others compare free-energy values to databases and also include noncanonical base-pairing and are therefore considered knowledge-based algorithms (such as MC-Fold|MC-Sym) [40]. Noncanonical base pairs can be formed by hydrogen bonds or stabilized by polar hydrogen bonds and even through interactions between C-H and O or N groups [41] and contribute interfering energies to the secondary structure prediction [40].

While secondary structures can be predicted based on the nucleotide sequence, the tertiary structures are far more complex. The aptamer shape is directly responsible for the binding affinity and specificity, and its 3D structure is collectively coded by information from the secondary structure [1]. Several algorithms for tertiary structure prediction of short NA molecules have been reported, but only a few of them are still accessible. In general, two main approaches exist for the prediction of tertiary structures: (i) prediction based on sequence analysis and (ii) prediction based on homologous sequence structures from databases. However, both approaches have disadvantages, which include the bad prediction of base pairing and pseudoknot formations and limited availability of known tertiary structures in databases, respectively. Furthermore, prediction of DNA and RNA is slightly different due to the additional oxygen atom in the RNA sugar, which allows for more hydrogen bond formation as well as the tendency of DNA to form double helix structures [42].

Nevertheless, it has to be noted that a group of researchers recently provided evidence that structural conversion between DNA and RNA molecules in silico resulted in almost identical hairpin formations and small fragment structures compared to experimentally gathered data [43]. Since research has far more interest in RNA molecules, significantly more prediction algorithms exist for RNA calculation. These include web-based algorithms, such as RNAComposer [44,45], BARNACLE [46],FARNA [47], MC-Fold|MC-Sym pipeline [40], and many others.

## 3. Historical Overview of Docking Algorithm Development

Interactions between aptamer and target are primarily based on polar and ionic interactions (extensively revised in references [9,14,48], and discussed in detail below), in addition to shape complementarity that results in binding properties comparable to monoclonal antibodies [6]. Predictions of these interactions are highly complex due to the flexibility and size of NAs as well as the already-mentioned chemical modifications, which lack experimentally determined structures as references in the databases. As an alternative, several different programs exist to tackle the problems as described in the following sections (Table 1).

While research focused on protein–protein interaction early on, computational approaches for protein–NA prediction and calculation have lagged behind [49,50]. With the discovery of ribozymes—small RNA molecules that exhibit enzymatic function—and post-transcriptionally regulated gene expression, interest in the field of RNA biology has fomented and grown exponentially [51,52,53]. Docking programs and algorithms have been designed to calculate protein–protein interactions and have been modified to also allow RNA sequences as ligands by implementing different parameters for the scoring functions without changing the core of their algorithms [54]. Docking algorithms fall into two main categories: (i) the machine learning algorithms, which predict molecule interactions based on sequence-based and/or structure-based information; and (ii) the template-based algorithms, which calculate interactions with information from known crystal structures [54].

First reports on the topic came from Katchalski-Katzir and colleagues, which employed rigid docking mode in combination with Fourier transformation to evaluate possible interaction sites in a six-dimensional-shape complementarity approach, termed GRAMM [55]. Rigid docking involves the putative binding of the three-dimensional NA on the receptor protein by considering the NA as a single immobile entity and only changing the overall coordinates by rotational and translational transformations. While this method was solely based on shape, newer algorithms, such as FTDock, have already implemented electrostatics and biochemical information, although they are still limited to rigid docking [56,57]. While the FTDock was designed for protein–protein interaction, nucleic acids can also be submitted. Moreover, the program has been further developed using a scoring mechanism and an algorithm for energy calculations, side chain optimization, and backbone refinement. Together with FTDock, they are described as 3D-Dock, an optimized docking program [58].

In parallel to these early approaches, the use of spherical polar Fourier correlation method, implemented in Hex docking, instead of using the Fast Fourier Transformation (FFT), has been shown to drastically accelerate the calculation speed [59]. Two additional convolution algorithms—DOT and DOT2—have also implemented Poisson–Boltzmann methods to better evaluate highly polar intermolecular interactions [60,61].

The program HADDOCK represented the first attempt to include not just the interaction information, but also allow flexible amino acid side chains, thereby increasing the quality of the docking models. Initially conceptualized for protein–protein interaction, HADDOCK nowadays also allows NA as input molecules [62]. HADDOCK’s algorithm has been refined to encompass the main challenges of protein–DNA docking, namely, the tendency of DNA to form double helix structures, together with the identification of DNA interaction sites [42]. While DNA often interacts with proteins via its major groove, further interaction with the minor groove must also be considered as the bigger DNA molecules represent one helical turn, which increases the possible binding sites [10]. PatchDock was developed with the aim of overcoming some of the calculation hurdles of existing shape complementarity docking algorithms. Although also geometry-based, the program calculates interaction sites with a higher efficiency using local feature matching instead of the usually applied six-dimensional transformation fitting [63].

While protein docking programs were optimized to also fit NA as input structures, first attempts for algorithms specially designed for NA–protein docking were reported around 2011. ParaDock, developed by Banitt and Wolfson, is an ab initio approach to calculate interactions solely based on the sequence of DNA and a rigid protein structure. While ParaDock still utilizes shape complementarity, the DNA structure is calculated from scratch in a flexible mode [64]. This is in contrast to most of the aforementioned software, which need the interface distant constraints as input [57,63,64].

The Bujnicki lab provided knowledge-based potentials for download to allow for protein–RNA docking in the same year. The potentials—named DARS-RNP and QUASI-RNP—use Decoys and quasi-chemical methods to describe the reference state and allow RNA and DNA docking, respectively [69]. Shortly afterwards, the same group presented a program called NPDock (short for nucleic acid–protein docking) which is based on the GRAMM algorithm implementing the DARS-and QUASI-RNP potentials and only allows rigid body docking, although considering the specific features of nucleic acids [53]. HDOCK, which was only recently released as a new algorithm focusing on “big molecule–big molecule” docking, operates on both template-based or template-free rigid docking mode, which also allows docking of molecules with unknown structures [65,66].

In contrast to rigid docking, flexible docking programs allow various conformations of the target molecules to find the state closest to the native form. Programs such as Gold [67], Autodock and Autodock Vina [68] had great success in small molecule drug discovery and have been employed to small and large NA docking. As a common characteristic, they all allow either full flexibility or rotamer-based search for both ligand and selected amino acids residues for docking in a determined binding pocket [67,68].

Although one might initially consider flexible docking as the best option, it should be kept in mind that calculation time exponentially grows with the increasing size of a molecule. Since aptamers are often initially composed of up to 70 nucleotides or even more, tertiary structures hold too much variability for flexible docking algorithms based on genetic (GA) or Lamarckian approaches to deal without high-performance computation (HPC) infrastructure. In this sense, binding site information from known structures is normally included in the docking procedure [65]. In cases where no crystal structure information on the protein complexes are available, NA binding site predictions can be carried out externally using other programs [70]. However, single strand DNA (ssDNA)-binding prediction algorithms are rarely available, which leads to the use of RNA-based counterparts as the most used option and results in another layer of uncertainty. Still, it must be mentioned that the atomic composition of RNA and DNA is highly similar and hence the usage of an RNA prediction algorithm for DNA binding sites have been shown [36,71].

## 4. Benchmarking and Quality Tests

Several studies report the use of the previously discussed algorithms in practical approaches to either predict aptamer binding to target proteins or to evaluate the quality of the docking calculations. In the following paragraphs, recent applications are presented and benchmark studies are emphasized.

In 2016, Torabi and colleagues utilized the HADDOCK program to predict the binding mode of retinol binding protein 4 (RBP4) and RBP4 binding aptamer (RBA) for further MD simulations. RBP4 is a biomarker used for type two diabetes pre-diagnosis, and the binding mode prediction with subsequent MD simulations was intended to elucidate the main aptamer–target interaction mechanics to support further aptamer design [72]. The group could discover the main forces driving the interaction between RBP4–RBA, shedding light on the mode of action, which highlighted this molecule’s inhibitory potential.

In a different study, HADDOCK, Autodock Vina, and Patchdock were applied as in silico controls to evaluate the binding affinity and specificity of aptamers against ERα. The aptamers were previously ab initio and in silico, designed using EREs as a template (see above, [29]). After validating the docking settings on a set of known protein–NA complexes against randomly generated RNA structures, the best docking protocol was used to predict aptamer–target interaction and select the best aptamer to bind ERα. The group could not only show that all docking algorithms concluded similar results but could also prove that the in silico predicted specificity and affinity in vitro [29]. Additional studies were designed especially to test existing docking programs, including the community-wide Critical Assessment of Prediction of Interactions (CAPRI) initiative [73,74] and a wide variety of benchmark databases.

For instance, Roberts and colleagues put their own DOT and DOT2 algorithm to the test using a set of four different protein–NA complexes [50]. They evaluated the prediction accuracy without any experimental knowledge compared to the structural data gathered from experimental procedures [50]. Similar studies were carried out for the HADDOCK algorithm, including several benchmark databases for protein–NA complexes [42]. Even more extensive efforts were made to benchmark the performance of the HDOCK algorithm on several known benchmark databases [65]. Additionally, many databases were created to offer opportunities for easy benchmarking of established docking programs and algorithms [49,75,76,77].

## 5. Evaluating the Quality of Docking

When evaluating the quality of the docking, some main ideas should be taken into consideration. The driving forces for NA–protein interaction are a milieu of van der Waals forces, hydrophobic interactions, hydrogen bonds, base stacking forces, and ionic interactions between amino acid side chains and either the phosphate groups or bases of NA [10,24]. Additionally, the importance of the shape complementarity provided by secondary and tertiary structures of NA and proteins, which contributes to the binding mode, should be stressed [24].

Ionic interactions commonly formed between positive amino acid side chains and the negatively charged DNA have been repeatedly proven important for NA–protein interaction [24,78,79,80,81]. Base stacking forces contribute to the stability of dsDNA but in particular support the binding of ssDNA to proteins involving stacking of bases and aromatic protein side chains [82]. At the same time, they are highly influenced by electrostatic interactions and van der Waals forces [83]. Hydrogen bonds, driven by dipole–dipole interactions, are especially formed more easily, and their energy can range between 4 and 40 kJ/mol [24,84,85]. Although their energy depends on pressure, angle, the distance between donor and acceptor (of at least 2.5 Å), and environment, the formation is not influenced much by surface structure [84,86]. The number of hydrogen bonds can be counted for each docking complex to measure the quality of the docking pose and the interaction itself, as already mentioned by Jones and colleagues [67] and discussed by Ahirwar et al. in 2016 [29]. Formation of hydrogen bonds has been demonstrated to be essential for biomolecular function and, hence, structure represents a key parameter of complex stability [87,88]. Despite the importance of π–π interaction, cation–π, and the overall electrostatic contribution, comparatively few studies have used other interactions as criteria for selection of NA docking poses [28,89]. As an example, Rabal employed clustering of docking results followed by evaluation of electrostatic and polar interactions between protein and RNA aptamers, similarly to ligand interaction fingerprints already employed as docking postprocessing of protein–ligand complexes [89].

Alternatives, such as consensus scoring, have been proposed to reduce the bias of single scoring functions [90], and the evaluation of true-positives retrieval rate from different programs can help. However, the notion that the highest dock score directly correlates with real ligand binding—and therefore with a biological effect—can be erroneous. Especially when applied to small molecules, docking analyses alone can create an inaccurate picture of ligand binding (an extensive discussion on the docking limitations was addressed by Chen [91]).

The knowledge of critical residues and, in this sense, the presence of respective interactions can bridge the virtual inferences and experimental results. In this sense, molecular modeling can give the structural perspective of the mutation effects while also benefitting from the experimental information.

Optimal prediction of native macromolecule conformations remains a challenge, especially since most of the docking approaches currently in use are based on a single rigid conformation. Despite the prolonged effort on the development of new docking algorithms, the ability to capture a full motion of the interaction between NAs and proteins is out of the scope of the technique, and more accurate predictions can be drawn from an integrated pipeline.

## 6. Molecular Dynamics Simulations Applied to Nucleic Acids (NA) and Protein–NA Interactions

Computational techniques can help to interpret protein–NA interactions and complement experimental results. To understand any protein–ligand interaction, the time dimension must be added to the snapshots of proteins frozen in crystal structures and docking poses. Molecular dynamics simulations can describe protein dynamics in detail, including the precise position of each atom at any instant in the simulation time—along with the corresponding energies—provided that at least one structure is known as a starting point. Briefly, molecular dynamics starts from the static structures experimentally determined, which represents the atom coordinates of macromolecules. These molecules are immersed in the solvent and have their positions updated along the simulation according to classical mechanic calculations of their interactions among themselves and with the solvent. The classical mechanic facet is represented by empirical force fields with optimized parameters for biological molecules. Furthermore, quantitative analysis of the conformational ensembles of the molecules during the long-enough simulations can reveal the thermodynamic properties of the biological system [92].

Simulation of nucleic acids and its interactions is an ever-growing field, which has lagged in comparison with the globular protein simulations. While the first protein simulations started in the early 1960s, the first simulation with nucleic acids dates from 1983 [93,94]. The reasons for this, as previously discussed by Mackerell [95], are the lack of NA–protein experimental complexes for validation of the studies, which also relied on inadequate models to treat electrostatic interactions and solvent.

Initial molecular dynamics simulation relied on the classical single point charge Coulombic model to describe electrostatic interactions, where the number of interaction partners was determined by a maximum distance cutoff. The exchange in the paradigm for electrostatic treatment—from the low cutoff Coulombic interaction model towards the Particle mesh Ewald method (PME)—was a milestone to NA simulations, since they are highly charged and nonglobular molecules. PME enabled the fast calculation of long-range interactions and its grid approach, instead of the classical static spherical cutoff, and improved the accuracy of NA simulation [96]. Additionally, modern HPC facilities enabled the use of explicit solvent representation as a standard option for NA’s simulations despite the higher computational cost compared to the implicit counterpart. HPC facilities, which initially relied on the algorithms Central Processing Unit CPU-parallelization, are now focused on the use of GPU-distributed calculations.

**Figure 1:**
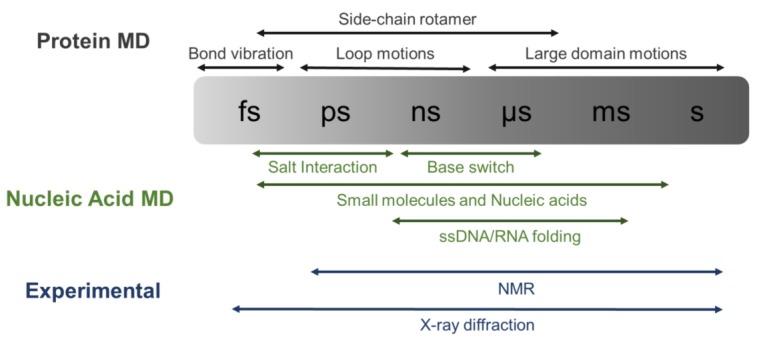
Timescale defines what can be observed by molecular dynamics simulation. The timescale of dynamic processes in proteins (Black), protein–nucleic acid complexes (Green), and the experimental methods (Blue) that can be observed from the different methods.

In terms of available force fields, specific torsions and bond’s parameters for protein–NA simulation were originally implemented on AMBER [97]. These parameters have been refined along the years through updates (AMBER ff94 up to ff99, and more recently the bsc1 or OL15 corrections [98]), besides classical force fields such as CHARMM, which have improved DNA representation parameters in the most recent version (CHARMM36) [99,100]. Most of the parameter corrections along the years focused on better representing NA-specific chemical features, which were poorly represented by other force fields, namely, anionic sugar−phosphate backbone [101] and the glycosidic dihedral chi angle for RNA and DNA [102]. However, a detailed discussion of the modified parameters is out of the scope of this review. While dsDNA counts on the stacking for the characteristic structural stability, as shown by long timescale simulations [99], the single-stranded NAs have a higher flexibility assuming multiple conformations. Molecular dynamics simulations can investigate the ssDNA distortion upon the protein binding considering specific NA interactions that arise from the free nucleotide bases [82]. Fast timescale simulations can define fluctuations around a defined state, representing properties such as interactions stability, amino acid flipping, or conformational change of small loops (Figure 1). Accordingly, Protein–double stranded (ds)NA complexes remain stable through an intricated network of hydrogen bonds and ionic interactions [103,104].

Molecular dynamic simulation of transcription factors on a microsecond scale showed that charged amino acids (side chains with NH_3_^+^) were responsible for this interaction. On the one hand, intermittent arginine–phosphate salt bridges with lifetimes of the order of hundreds of picoseconds [105,106], which is in line with NMR studies [107], were relevant for general dsDNA binding (Figure 1). The importance of explicit solvents to represent those interactions cannot be understated, since Arg–Phosphate indirect interactions, water-mediated, are as relevant as the direct bridge [108]. On the other hand, complementarily, interactions between arginine and NA bases helped to elucidate the differential binding to the consensus sequences.

Short simulations of the *Corynebacterium pseudotuberculosis* cold shock protein A bound to an aptamer molecule were performed by Caruso [109]. They have shown that ssDNA can also rely on intermittent nonspecific hydrogen bond interactions, in addition to the salt-bridges, to confer complex stability [109]. Interactions between aromatic amino acids and the free nucleic acid bases contributed to an overall enthalpy energy gain. Protein–aptamer complexes simulation on a short scale of few nanoseconds were able to suggest the stability of the aptamer secondary structure; however, induced-fit effects would require longer simulations and extensive sampling [72,110,111].

It should be noted, however, that protein dynamics are characterized not only by the timescale of the atomic fluctuations (short simulations of few nanoseconds) but also by the amplitude and the directionality of the fluctuations [112]. Long range conformational transitions, despite rarely occurring in short MDs, are relevant because many biological processes—including protein–protein and protein–NA interactions—occur on the timescale of microseconds. Although it is possible to routinely perform microsecond-length simulations of fully solvated atomistic nucleic acids with reliable convergence [113,114,115], unfortunately, protein–NA dynamics on the microsecond-to-millisecond timescale cannot routinely be performed in most laboratories.

New approaches that either simplify force fields or the conformational sampling have been developed along the years to overcome these limitations including, for instance, normal mode analysis (NMA) [116,117] and Markov state models (MSM) [118]. NMA can describe slow large-amplitude motions of the proteins, sometimes restrained to the Cα atoms only, using calculated low frequencies of the vibrational normal modes. Due to the simplified nature of NMA calculations relying on the intrinsic harmonic oscillations of protein, this method is limited to investigating fluctuations around a specific conformation. In this sense, the transcription factor Catabolite Activator Protein (CAP) has been showcased as an example of protein–NA complex treated by NMA [119]. Despite the clear influence of the DNA in the different simulated systems, by comparing NMA calculated from structures cocrystallized with and without the nucleic acid, the DNA was not explicitly considered in the NMA calculations.

On the other hand, MSM relies on comprehensive sampling of simulated trajectories to generate a network model. This network can allocate the conformational space into discrete states and suggest the kinetic aspect from the transition probabilities between the states. MSM has the advantage of statistically approaching the diversity of conformations and—even when the amount of collected data is poor—MSM can guide data collection, e.g., by selecting new MD simulation’s starting points.

For instance, thrombin and the thrombin-binding aptamer (TBA), with the sequence 5′-GGTTGGTGTGGTTGG-3′), which is under investigation as an anticoagulant drug, have been employed as models for better understanding the effect of long simulations on both the aptamer folding/unfolding process and interaction with the protein [120]. Crystal structures of this complex reveal TBA-binding in the fibrinogen binding site (also called exosite-I), preventing thrombin cleavage by fibrinogen. Long simulations of thrombin, complexed with TBA, showed a reduced number of protein conformation states towards a single population with reduced flexibility in the surface loops. Complementarily, simulations of thrombin without aptamer-binding had a larger number of unique conformations, which became inaccessible after aptamer-binding [120]. Also, thrombin has a second RNA-addressable site—the exosite II—which can be exploited to inhibit the thrombin-dependent platelet activation [121]. Inhibition of both sites can decrease procoagulant activity synergistically, which suggests an allosteric mechanism.

In conclusion, both NMA and Markov models have had great success in reproducing the conformational diversity of protein and NA states individually; however, they must still achieve the popularity and precision that MD currently have, especially concerning Protein–ssNA applications. MD of free TBA followed by the inference of MSM revealed an unfolding process with interconnected multistate intermediates, which can provide important insights into the diverse conformational set of this important aptamer [122].

In terms of force field simplification, simulations employing coarse-grained models were employed for studying large-scale conformational changes in free RNA molecules and riboswitches [123,124,125]. Coarse-grained models simplify groups of individual atoms as a single entity, encompassing the main properties of the parts. By decreasing the number of simulated elements and therefore the degrees of freedom, there is a boost in simulation performance in detriment to the detailed system description. Currently, there is a range of tools involved in the single stranded RNA (ssRNA) structure prediction and validation [126]; however, a valid prediction pipeline for ssDNA has recently been developed [43]. Jeddi and Saiz employed an approach to integrating the secondary structure information of ssDNA into the 3D ssRNA modeling software available, followed by the conversion of the final 3D structure into its ssDNA equivalent [43]. Additionally, they showed that atomistic MD simulations could be used to improve the correlation between the predicted and native structures, mainly on hairpin-like structural motifs. Liu and collaborators investigated the stability of small molecules ligand binding pose by MD [127,128]. The proposed binding mode encompassed real ligands and decoys both self-docked in the original structure and cross-docked into homologs and homology models.

In general, simulations should reproduce known experimental data or complement its interpretation; however, on one hand, there is no experimental equivalent for the MD ability to capture protein motions in so many different scales and on the other hand, the reproducibility is a recurrent issue (these issues have been extensively discussed by [129]). NMR spectroscopy is a technique capable of exploring some of the protein motions. Specifically, NMR experiments such as nuclear spin relaxation and relaxation dispersion enable the evaluation of motions at pico–nanoseconds and micro–milliseconds timescale, respectively [130]. Accordingly, given that enough details are provided, MD simulation, as well as any experimental procedure, should be reproducible; however, documentation on important details, such as force field version and parameters adjustment, are often lacking. Additionally, there are several technical limitations to the use of MDs which will be described in the following section. Molecular dynamics are stochastic in nature and highly dependent on the initial state since the simulation time often is insufficient to overcome potential energy barriers and sample diverse conformation changes. In this case, simulation represents the atomic changes within the microenvironment around the initial state and its respective thermodynamic ensemble. Solvent representation in simulations is important not just for the overall protein folding but also, as previously mentioned, to model the electrostatic interactions since most of the salt bridge interactions are mediated by water networks. However, often solvent and ion numbers are reduced to save computational power, which decreases the ability to accurately represent the water shell around the complexes [131].

Finally, although experiments can determine what is moving and how fast, molecular dynamics simulations can answer why things move because the underlying forces and corresponding energies are included in the simulation. The resulting predictions can inspire new experiments trying to answer how the nucleic acids interact with the proteins and therefore, despite the aforementioned limitations, MD can still be employed as an important tool as long as timescale and parameter are considered.

## 7. Conclusions and Future Perspectives

Cheatham highlighted the importance of sampling for understanding nucleic acids properties, particularly when the binding energy estimation is concerned [132]. Herein, we presented the importance of nucleic acids in recent biological and biomedical research and gave an introduction to discovery and the development of functional NAs, including SELEX and alternative in silico approaches. We further gave an overview of in silico modeling approaches of NAs and proteins and especially discussed the importance of appropriated scale simulations to evaluate the stability between protein and large nucleic acids (mostly aptamers) in the complex. We also highlighted the importance of post-processing, such as evaluation of electrostatic and hydrogen bond interactions after docking. However, even after MD simulations, a molecular docking result can just show that the nucleic acid binds well to the target protein. Therefore, it is not advisable to overinterpret the docking results before other experimental validations have been performed. In terms of docking itself, new alternative algorithms are likely to be developed in the near future, together with machine learning techniques, which can improve the tridimensional structure prediction for the aptamers, therefore filling the current structural gap.

The current state of the art shows that long simulations with dsDNA and even protein–dsDNA are possible. However, simulations of ssNA remain a challenge, since the importance of base-stacking interactions versus exposure to explicit solvent is still unbalanced, which could be solved with new parameters for the current force fields. Additionally, several factors that can influence the kinetics of protein–nucleic acid interactions, such as viscosity and pH of a solution, and changes on the protonation state of amino acids are out of the scope of classical molecular dynamics. Recently, the use of polarizable force fields on simulations have been discussed as the next generation step for molecular dynamics simulations, which would not only address the mentioned limitations but also better represent base stacking [133]. Finally, the ever-growing computational power, which initially benefited from parallelization, has nowadays culminated in the advanced GPU high-performance centers. We expect that large-scale simulations for protein–NA complexes will shed a light on the interactions and can become an integral part of this field of research.

## Figures and Tables

**Table 1:** A chronological overview of docking algorithms according to the mode of action.

Algorithm	Milestone	Reference
GRAMM	Rigid docking, six-dimensional shape complementarity; fast Fourier transformation	[55]
FTDock	Implementation of electrostatics and biochemical information	[56,57]
3D-Dock	Additionally, energy calculations, side chain optimization, and backbone refinement	[58]
Hex	Spherical polar Fourier correlation method	[59]
Dot/Dot2	Implementation of Poisson–Boltzmann methods	[60,61]
HADDOCK	Flexibility of amino acid side chains	[62]
PatchDock	Local feature matching instead of six-dimensional transformation fitting	[63]
ParaDock	Shape complementarity but flexible NA structure prediction	[64]
NPDock	Rigid body docking while considering the specific features of NA	[53]
HDOCK	Docking between two big molecules; template-based and template-free rigid docking mode	[65,66]
Gold	Full flexibility or rotamer-based search for both ligand and selected amino acids residues; docking in a determined binding pocket. Presents a range of different scoring functions, from machine-learning-based to physicochemical-based ones	[67]
Autodock Autodock Vina	Full flexibility or rotamer-based search for both ligand and selected amino acids residues; docking in a determined binding pocket. Energy-based scoring function and ability to handle surface pockets	[68]
